# Data After Death: Post-Mortem Ethics of Burn Patient Records and Images

**DOI:** 10.1093/jbcr/irag097

**Published:** 2026-06-13

**Authors:** Joshua Khorsandi, Brian Mansoury, Liahm Blank, Abu-Bakr Ahmed, Samir Alkhouri, Kathryn Cataldo, Joshua MacDavid

**Affiliations:** Kirk Kerkorian School of Medicine at University of Nevada Las Vegas, Las Vegas, NV 89106, United States; Kirk Kerkorian School of Medicine at University of Nevada Las Vegas, Las Vegas, NV 89106, United States; Kirk Kerkorian School of Medicine at University of Nevada Las Vegas, Las Vegas, NV 89106, United States; Kirk Kerkorian School of Medicine at University of Nevada Las Vegas, Las Vegas, NV 89106, United States; Kirk Kerkorian School of Medicine at University of Nevada Las Vegas, Las Vegas, NV 89106, United States; Department of Plastic and Reconstructive Surgery, University of Nevada Las Vegas, Las Vegas, NV 89106, United States; Department of Plastic and Reconstructive Surgery, University of Nevada Las Vegas, Las Vegas, NV 89106, United States

**Keywords:** burn care, medical photography, post-mortem privacy, posthumous consent, artificial intelligence, ethical governance

## Abstract

Burn care frequently relies on extensive documentation, including graphic photographic images and detailed clinical records. While these materials are essential for diagnosis, treatment planning, and research, their use after a patient’s death raises complex ethical questions. The emergence of new technologies such as artificial intelligence training, alongside the increased visibility of burn images in education and public health campaigns, challenges traditional notions of confidentiality and consent. This narrative review examines the ethical boundaries of using burn patient records and images post-mortem, with a focus on emerging concerns around digital remains and posthumous consent. A narrative review of peer-reviewed literature, professional guidelines, and position statements published between 2000 and 2025 was conducted. Sources included PubMed, Scopus, and Google Scholar, using search terms such as “burn injuries,” “medical photography,” “post-mortem consent,” “digital remains,” and “medical ethics.” Relevant publications addressing clinical practice, teaching, research, and social media use in burn care were synthesized to identify key ethical themes and gaps. The literature reveals that while ethical frameworks for consent, privacy, and confidentiality are well established during life, guidance becomes inconsistent once the patient has died. A small but growing body of scholarship identifies posthumous privacy as an emerging domain of bioethics. Across studies, concerns included dignity after death, risks of re-identification on digital platforms, and the absence of explicit patient directives regarding posthumous use of images and data. Current medical guidelines provide minimal direction, leaving ambiguity for clinicians and researchers. The ethical use of burn patient images and records after death remains underexplored, particularly in the context of artificial intelligence training datasets and social media awareness campaigns. The absence of consensus underscores the need for professional societies to establish clearer policies and protocols that honor patient dignity beyond life. Establishing standards for posthumous consent will help clinicians, educators, and researchers navigate the evolving landscape of digital medicine responsibly.

## INTRODUCTION

Burn care is a profoundly visual specialty in which surface appearance, color, depth, and anatomical context shape nearly every diagnostic and therapeutic decision. Photographic documentation has therefore become routine in modern burn units to supplement written descriptions, support longitudinal assessment, and enable multidisciplinary communication, particularly as digital imaging and smartphone technology have proliferated. Recent institutional reviews in burn centers demonstrate the centrality of wound photography for tracking healing trajectories, informing operative timing, and supporting education and research.^1^ Telemedicine programs using burn images transmitted from referring hospitals or mobile devices have been shown to influence transfer decisions and improve triage, underscoring how visual data now circulate well beyond the bedside.^2^

Parallel developments in clinical photography and digital record-keeping have raised persistent ethical questions regarding consent, confidentiality, and secondary uses of images. In dermatology and other visually oriented specialties, commentators have described how ubiquitous smartphone cameras, electronic medical records, and cloud storage complicate traditional expectations about who controls clinical photographs and for what purposes they may be used.^3–5^ Ethical analyses and practice guidelines emphasize the need for explicit consent, secure storage, and role clarity around educational and publication uses.^6^ Yet these discussions typically presuppose a living patient who can authorize or refuse particular uses of their image.

At the same time, burn care increasingly intersects with networked digital ecosystems in which images circulate far beyond controlled clinical environments. Burn wound photographs now appear in ICU diaries, which patients and families describe as important tools for reconstructing amnestic periods of critical illness.^7^^,^^8^ Social media and online advocacy platforms have amplified burn survivor narratives and images, blurring boundaries between personal testimony, public education, and clinical disclosure and prompting calls for more robust guidance on the sharing of medical images in public forums.^9–11^

Concurrently, artificial intelligence (AI) and machine learning have begun to rely heavily on clinical images, including burn photographs, for automated segmentation, depth estimation, and outcome prediction. Systematic reviews document rapid growth in burn-related AI applications, and recent work describes specialized burn image datasets and advanced convolutional architectures trained on thousands of wound photographs.^12^ This data-intensive turn has renewed attention to re-identification risks and to the provenance of training sets assembled from clinical archives or scraped from public platforms, often without clear, prospective consent for such use.^13–15^

Against this backdrop, the ethical status of burn images and records after a patient’s death remains strikingly underarticulated. In general medical ethics, professional guidance affirms an obligation to protect confidentiality postmortem, subject to limited exceptions.^16^^,^^17^ Legal regimes such as the HIPAA Privacy Rule in the United States extend protections to decedents’ health information for 50 years after death, while leaving substantial discretion regarding research and archival uses.^18^ Emerging scholarship in philosophy, law, and information ethics argues that “post-mortem privacy” and “digital remains” deserve normative protection akin to bodily remains, particularly as personal data outlive biological death and can still be used in ways that affect reputations and relationships.^19–21^ However, burn-specific literature has only recently begun to address ethics systematically and continues to focus largely on decision-making, pain, and end-of-life care rather than on data and images.^22^^,^^23^

It is important to note, however, that strong claims about posthumous privacy, autonomy, or rights remain contested. Some philosophers and legal theorists argue that deceased persons cannot be harmed in the same way as living persons because they no longer have experiential interests, welfare interests, or agency in the ordinary sense.^24^ From this perspective, restrictions on post-mortem use of health data may require justification not primarily by reference to the rights of the dead, but by reference to other concerns, including prior promises made to patients, respect for ante-mortem preferences, effects on surviving family members, public trust in medicine, and the risk of deterring living patients from consenting to clinical documentation or research. Other data ethics approaches emphasize that posthumous medical data may have substantial social value for research, quality improvement, and future patient care, meaning that privacy-based concerns must be balanced against potential benefits rather than treated as absolute barriers to use.^20^ This review therefore does not assume consensus that deceased persons hold privacy rights equivalent to living persons; rather, it identifies how disagreement in the broader ethics literature leaves clinicians, researchers, and institutions without clear guidance for image-specific post-mortem use.

This review focuses on several practical use cases in which posthumous use of burn patient images and records may raise distinct ethical concerns: (1) continued use of clinical photographs for internal education, case review, quality improvement, or clinician training after a patient has died; (2) use of archived burn images and clinical data for research or AI model development, including datasets created before AI-related secondary uses were foreseeable; (3) public or semi-public dissemination of burn images through journals, conferences, social media, advocacy campaigns, or fundraising materials; and (4) transfer, retention, or sharing of images with families through ICU diaries, memorial materials, or other personal archives. These use cases are not ethically identical, but each raises questions about posthumous consent, dignity, identifiability, family interests, and the limits of existing medical photography and confidentiality guidance.

**Figure 1:**
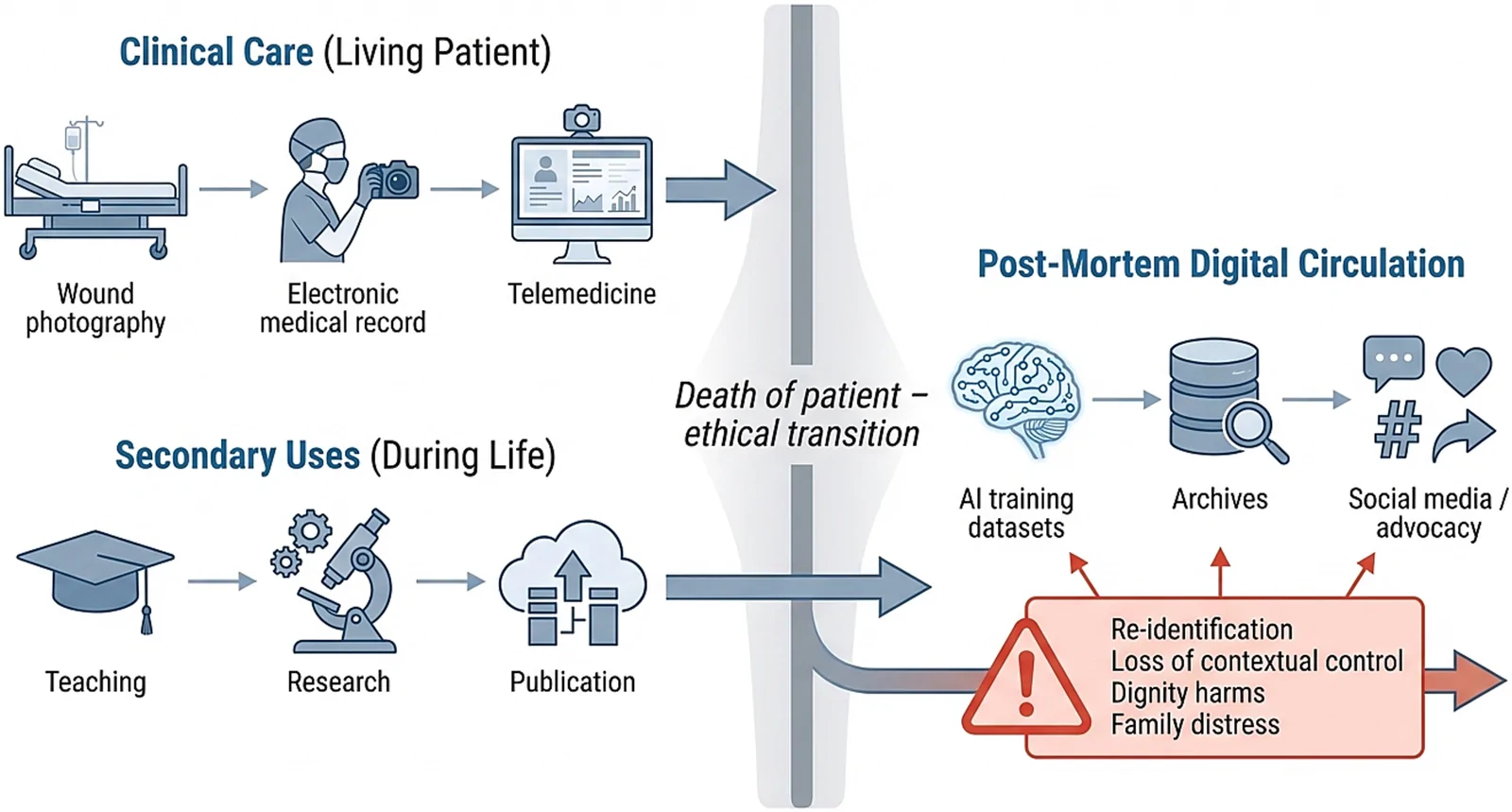
Lifecycle of Burn Patient Images Across Life and Death. Burn photographs originate in clinical care but may later circulate through education, research, social media, and AI systems after death, where ethical oversight and consent become less clearly defined. Image created by authors using FigureLabs.

This narrative review synthesizes peer-reviewed literature, professional guidelines, and position statements from 2000 to 2025 on burn documentation, medical photography, posthumous privacy, digital remains, AI datasets, and post-mortem use of clinical records and images. Guided by the emerging concept of posthumous privacy, it examines how ethical frameworks for consent and confidentiality shift after death, analyzes risks of re-identification in digital spaces, evaluates the near absence of explicit patient directives for post-mortem use of burn images and data, and assesses the adequacy of existing professional guidance for clinicians, researchers, and data scientists working in burn care. This review is intended as a gap-focused synthesis rather than a definitive legal or ethical governance proposal. The goal is to identify domains requiring further ethical, legal, clinical, and professional consensus rather than to prescribe a complete governance framework. Figure 1 maps the lifecycle of burn patient images and clinical records from bedside documentation through post-mortem digital circulation, illustrating how ethical oversight and consent clarity often diminish as visual data move beyond the clinical context.

## METHODS

This narrative literature review was designed to map conceptual, ethical, and practical discussions related to post-mortem use of burn patient records and images rather than to estimate effect sizes or aggregate quantitative outcomes. Consistent with methodological guidance for narrative and ethical reviews, the approach prioritized breadth, theoretical richness, and contextual interpretation over exhaustive retrieval of all empirical studies.

Electronic searches were conducted in PubMed, Scopus, Web of Science, and Google Scholar for publications between January 1, 2010 and October 31, 2025. Core search terms combined controlled vocabulary and keywords related to burn care (“burn injuries,” “burn unit,” “burn wound,” “burn photography,” “teleburn,” “burn ICU diary”), clinical imaging (“medical photography,” “clinical photography,” “wound photography”), post-mortem and privacy concepts (“post-mortem consent,” “posthumous privacy,” “decedent confidentiality,” “digital remains,” “digital legacy”), data ethics (“medical ethics,” “bioethics,” “data donation,” “posthumous medical data donation”), and artificial intelligence (“machine learning,” “deep learning,” “AI training datasets,” “medical imaging re-identification”). Reference lists of key articles and editorials were hand-searched to identify additional scholarship.

Inclusion criteria encompassed peer-reviewed empirical studies, conceptual or normative articles, systematic and narrative reviews, law review and ethics journal pieces, and book chapters that addressed at least one of the following domains: documentation or photography in burn care; ethical or legal analysis of medical photography or clinical images; post-mortem confidentiality or privacy of health information; digital remains or digital afterlife; AI and large-scale medical imaging or electronic health record datasets; and posthumous consent or medical data donation. Professional guidelines and position statements from relevant organizations, including the American Medical Association and health privacy regulators, were also reviewed when they explicitly addressed post-mortem confidentiality or image use. Publications not available in English, comment threads without substantive analysis, and purely technical AI papers with no consideration of ethics or consent were excluded.

Titles and abstracts were screened for relevance to burn care or to broader questions of medical image and data use after death. Articles were then read in full and coded inductively for ethical themes, including concepts of dignity, privacy, consent, ownership, familial interests, re-identification risk, and digital legacy. Particular attention was paid to whether the living/deceased distinction altered authors’ ethical conclusions, whether burn-specific considerations were articulated, and whether explicit guidance was offered for clinicians, researchers, or data custodians. Themes were iteratively refined through constant comparison across burn-specific and general literature, and are presented narratively as overlapping domains rather than mutually exclusive categories. Because this review aims to synthesize normative arguments and emerging concerns, no formal quality appraisal or risk-of-bias assessment was undertaken, although empirical studies were interpreted in light of their design limitations and context. In total, 42 publications met inclusion criteria and were synthesized to inform the ethical analysis presented in this review.

## RESULTS

### Burn documentation, medical photography, and visual culture in burn care

Burn literature shows photographic documentation is embedded in clinical workflows: wound photography supplements written notes, supports serial assessments, and informs operative planning.^1^ Pediatric burn centers report high use of medical photography, especially for large total body surface area burns and complex reconstructions, with images integrated into the electronic record and used for case conferences and teaching.^25^ Telemedicine initiatives similarly depend on digital images from referring hospitals or smartphones to guide triage and transfer decisions; early evaluations suggest photographs can change clinicians’ assessments of burn size/severity and sometimes reduce unnecessary transfers.^2^

Burn ICU diaries reflect a related practice: photographs embedded in diaries kept by clinicians and families to document critical illness. Patients and nurses describe images as “filling in the blank spaces” of amnestic periods and helping patients interpret altered bodies during recovery, while also noting their potentially shocking nature.^7^^,^^8^ Similar findings in general ICU populations link photo-diaries to improved psychological outcomes and stronger narrative continuity after discharge.^26^ These practices underscore that burn photographs can become part of personal and family archives with emotional and memorial functions that may persist after death.

Across specialties, ethical analyses emphasize that digital photography introduces risks of unauthorized copying, onward sharing, and loss of contextual control.^3^^,^^4^ Patient attitude surveys generally support photography when clearly tied to care, but show greater ambivalence about research or teaching uses—especially for intimate or identifiable images. Commentators therefore stress purpose-specific consent (use, storage, and future uses) rather than bundling photography into general treatment consent, and highlight the sensitivity of graphic or disfiguring images.^5^^,^^6^

### Social media, public dissemination, and blurred boundaries

Social media has become a major pathway for clinical images to circulate beyond traditional professional channels. Commentaries in gastroenterology, pathology, and surgical ethics describe physicians sharing photographs on platforms such as Twitter, Instagram, and Facebook for teaching, case discussion, and professional branding, often without clear institutional oversight.^9^^,^^10^ These authors emphasize that consent for journal publication does not straightforwardly translate to broader, more permanent, and less controllable online dissemination. Analyses from global health and nursing similarly warn that posting patient photographs from humanitarian or mission settings can slide into voyeurism or “poverty porn” when images are used for fundraising or personal branding, even when nominal consent is obtained.^27^

Professional guidance relevant to online sharing includes the American College of Surgeons’ recommendation to obtain explicit consent before posting any patient-identifiable image and to avoid content that could be exploitative or disrespectful.^28^ The AMA Code of Medical Ethics reiterates that confidentiality obligations apply on social media and that de-identification must be robust enough to prevent re-identification by patients or acquaintances.^29^ However, burn-specific frameworks remain sparse despite high public interest in burn survivor narratives and widespread patient-led advocacy and awareness campaigns.

The post-mortem dimension of social media image sharing is rarely addressed in clinical guidance. Case examples outside burn care—such as unauthorized sharing of autopsy images or viral circulation of graphic trauma photographs—illustrate the distress such breaches can cause and how digital images can outlive both patients and initial clinical intentions.^10^^,^^30^ Given their often graphic and stigmatizing nature, burn images may be especially vulnerable to secondary circulation when used in awareness campaigns or shared online by clinicians and survivors.

### Artificial intelligence, imaging datasets, and re-identification risk

Burn-related AI research has expanded rapidly. Previous studies describe growing use of machine learning to predict burn depth, grafting need, length of stay, and mortality using clinical variables and images.^31^ Image-focused studies report convolutional neural networks and generative adversarial networks for segmentation and classification, often trained on curated datasets of color burn photographs from single centers or multi-institution aggregates.^32^ Technical reports also describe “Extended Burn Image Segmentation” datasets and architectures such as BurnsNet intended to standardize labels and improve generalizability.^33^

These developments intensify questions about whether consent for AI training is adequate and whether de-identification is sufficient. General medical imaging scholarship increasingly rejects the assumption that simple facial cropping or removal of obvious identifiers fully protects anonymity; reconstructed facial images from MRI scans can be matched with publicly available photographs using face-recognition methods, suggesting de-identified images in open repositories may be re-identifiable when linked with auxiliary information.^34^

This risk is likely to expand as AI systems become more capable of identifying individuals from partial or nontraditional biometric cues. In burn photography, re-identification may not require an unredacted face. Partial facial contours, ears, hands, tattoos, scar distribution, body habitus, posture, and, in video-based documentation, gait may all function as identifying features when combined with external photographs, social media profiles, or other auxiliary datasets.^35^^,^^36^ These concerns are especially important in burn care because images often include large body surface areas, distinctive wound patterns, reconstructive outcomes, and contextual metadata such as age, sex, injury mechanism, date, or treating institution. As a result, “de-identification” should not be treated as a fixed technical achievement, but as a context-dependent and time-sensitive risk assessment that may change as biometric recognition tools improve.

In burn-specific AI publications, descriptions of consent and governance are often minimal. Many papers note IRB approval and “de-identification” without clarifying whether patients were informed about AI development or whether post-mortem withdrawal or preferences were considered.^37^ Some datasets include whole-body or facial images in which scars, tattoos, or other features could remain recognizable to family members even without explicit identifiers—particularly when combined with metadata such as age, sex, and injury mechanism—and few papers acknowledge that images of deceased patients may persist within training sets and be replicated or transformed long after death.^38^

More broadly, controversies surrounding large-scale image–text datasets used to train generative models underscore ethical and legal concerns when images are scraped from the web without consent, including images of children and other vulnerable groups.^39^ While not burn-specific, these cases illustrate how digital traces—including those created for advocacy or memorialization—can be absorbed into opaque AI pipelines without permission, raising concerns about dignity, exploitation, and “data of the dead.”

### Posthumous privacy, digital remains, and the informational body

Scholars outside burn care have developed accounts of posthumous privacy and digital remains. Philosophers and legal theorists argue that individuals can be harmed after death through reputational damage, betrayal of confidences, or violations of expressed preferences, and that informational traces of the dead—the “informational body”—deserve protection akin to physical remains.^19^^,^^30^ Digital remains—profiles and data persisting after death—are also studied in cyberpsychology, human-computer interaction (HCI), and information ethics as frameworks for grief, memorialization, and the political economy of the “digital afterlife industry”.^40^

In health data ethics, Bak and Willems propose “contextual exceptionalism after death,” arguing deceased persons retain a prima facie moral right to privacy as informational entities grounded in respect for the dead and public trust in research.^41^ They note that guidance has focused largely on genetics, leaving other health data—including imaging and clinical records—under-regulated. Pearce’s analysis of posthumous medical data donation (PMDD) draws analogies to organ donation and examines whether ante-mortem consent should be required for using decedents’ medical data in research, arguing robust consent mechanisms may be needed to align PMDD with evolving data protection norms.^20^ Ashley extends similar reasoning to consumer data, arguing privacy rights should extend posthumously to fulfill modern privacy legislation.^21^

Ethical literature on the secondary use of biological and genetic materials provides an important analogue for burn images and records. Like clinical photographs, biospecimens and genetic data are often collected in clinical contexts but may later acquire new research value through technologies or research questions that were not foreseeable at the time of collection. Biobanking scholarship has emphasized recurring concerns about broad consent, donor control, privacy, future unspecified uses, withdrawal, and potential effects on biological relatives or communities.^42^^,^^43^ These concerns are particularly relevant after death, when the original donor can no longer clarify preferences or respond to new secondary uses. A systematic review of stakeholder perspectives on post-mortem use of genetic and health-related data found that many consent frameworks do not clearly address post-mortem use, creating uncertainty after participants die.^44^ Although burn photographs are not biological samples, they raise parallel concerns because they can remain identifiable, emotionally sensitive, and scientifically valuable long after the original clinical encounter.

These frameworks align with longstanding medical debates about confidentiality after death. The AMA’s Council on Ethical and Judicial Affairs has held that patients are entitled to the same respect for confidentiality after death as in life, with limited exceptions.^16^^,^^17^ HIPAA’s 50-year rule likewise recognizes enduring interests of descendants and families.^18^ However, these instruments rarely address image-specific risks, AI training, or the dignity implications of burn photographs that may depict naked, sedated, or disfigured bodies.

### Absence of explicit patient directives and fragmentation of guidance

Across burn-specific and general medical literature, few mechanisms allow patients to specify preferences about post-mortem use of clinical images and records. Burn photography consent typically addresses clinical care, possible teaching and research uses, and sometimes publication, but rarely clarifies what should happen after death, whether families can veto or authorize new uses, or whether images may be retained indefinitely for AI development or future clinician training.^3^ ICU diary studies similarly note diaries and photographs are often given to patients or families without structured discussion of storage, sharing, or handling if the patient dies, despite diaries’ hybrid clinical–personal status.^7^^,^^8^

PMDD discussions suggest data-donor frameworks as one route to honoring posthumous preferences, but these initiatives remain largely theoretical and focus on electronic health record (EHR) and genomic data rather than highly sensitive images.^20^ Bak and colleagues also argue for context-specific governance to determine when decedent data may be used without explicit consent, yet current regulatory frameworks provide few criteria beyond broad appeals to public interest or scientific value.^41^

Within burn care, ethics scholarship has addressed decisional capacity, treatment refusal, and end-of-life care, but has not systematically extended to data and image use. Reviews and case discussions in the AMA *Journal of Ethics* and the *Journal of Burn Care & Research* emphasize conflicts among autonomy, beneficence, and justice in high-risk decisions and socially complex care, but rarely consider how documentary and photographic traces might be used after death.^22^^,^^45^^,^^46^ This gap is especially salient given the emotional weight images may carry for bereaved families and the risk of secondary digital circulation in educational or advocacy settings. Table 1 summarizes how major legal, professional, and bioethics frameworks address (or fail to address) post-mortem use of clinical images, highlighting persistent gaps relevant to burn photography and AI training.

**Table 1:** What Current Frameworks Say About Post-Mortem Confidentiality and Where They Fall Short for Burn Images and AI

**Framework/source category**	**What it clearly supports**	**What it does not clearly resolve (burn/AI-specific gaps)**	**References**
US legal privacy rule for decedents	Decedent health information remains protected for a defined post-death period; disclosure is constrained and research access may occur under specific conditions	Whether AI model training counts as a distinct “use,” and what governance is appropriate for image archives and dataset sharing beyond baseline compliance	^18^
Professional medical ethics guidance	Duty of confidentiality generally persists after death; social media and clinical image sharing require strong privacy protection and careful consent	How to handle family requests vs patient preferences; whether “publication consent” covers online permanence, secondary sharing, or AI dataset inclusion	^16^ ^,^ ^17^^,^ ^27^^,^ ^28^
Posthumous privacy + PMDD scholarship	Ethical arguments for protecting “informational remains,” with context-sensitive governance and calls for structured mechanisms for posthumous preferences (analogous to donation/legacy planning)	Operational details for burn photography workflows (eg, risk tiering for graphic images; rules for tattoos/scars; controlled-access dataset standards)	^20^ ^,^ ^21^^,^ ^38^^,^ ^41^

Abbreviations: AI = artificial intelligence; PMDD = posthumous medical data donation; US = United States.

## DISCUSSION

This review reveals a pronounced disjunction between the centrality of visual and documentary practices in contemporary burn care and the relative absence of explicit ethical frameworks governing the post-mortem use of these materials. While burn units have embraced photography, telemedicine, and increasingly AI-driven image analysis for legitimate clinical and research purposes, the literature indicates that consent practices, professional guidance, and regulatory frameworks continue to focus primarily on living patients and on more traditional forms of data sharing.

The practical concern, therefore, is not simply that burn images persist after death, but that they may be reused in different contexts with different ethical risks. Internal quality improvement, clinician education, AI training, public awareness campaigns, and family-held image archives each involve different audiences, degrees of identifiability, consent expectations, and potential effects on surviving relatives. The gaps identified in the literature are most clinically relevant when understood in relation to these specific use cases.

From a conceptual standpoint, the emerging discourse on posthumous privacy and digital remains offers important resources for addressing this gap. One strand of the posthumous privacy literature argues that individuals may persist after death as “informational entities” whose reputations and relationships can still be affected by disclosures, a view that aligns with longstanding medical commitments to confidentiality and respect for persons.^19^^,^^41^ However, this is not a settled position across philosophy, law, or data ethics, and competing views give greater weight to public benefit, research value, family interests, or the idea that duties after death are grounded in promises and social trust rather than direct rights held by the deceased.^20^^,^^24^^,^^47^ When applied to burn images and records, this perspective suggests that the ethical justification for protecting confidentiality does not evaporate at the moment of death; rather, the focus shifts from protecting future bodily or economic interests to safeguarding dignity, narrative integrity, and the interests of survivors.

At the same time, there are powerful reasons to continue using burn images and records after death for education, quality improvement, and research. Historical case collections, atlases, and image-based teaching resources have long relied on photographs of deceased or severely injured individuals to train surgeons and emergency physicians, and AI models may one day enable more accurate triage, personalized prognostication, and resource allocation in burn disasters.^23^ Appeals to beneficence and justice thus support ongoing use of decedent data to improve care for future patients. The key ethical challenge is not whether post-mortem use is permissible in principle, but under what conditions and with what safeguards it respects the interests of the dead and the living.

Existing legal and professional frameworks offer partial guidance. HIPAA’s 50-year protection period for decedent health information and the AMA’s insistence that confidentiality generally survives death establish a presumption against unrestricted post-mortem disclosure.^16^^,^^18^ However, these frameworks leave unclear whether including a deceased patient’s burn photographs in internal AI training constitutes an authorized “use,” whether broad lifetime research consent remains morally valid for unforeseen technologies and dissemination channels, and how clinicians should resolve conflicts between the patient’s privacy preferences and family requests (eg, using graphic images for public advocacy).

**Figure 2:**
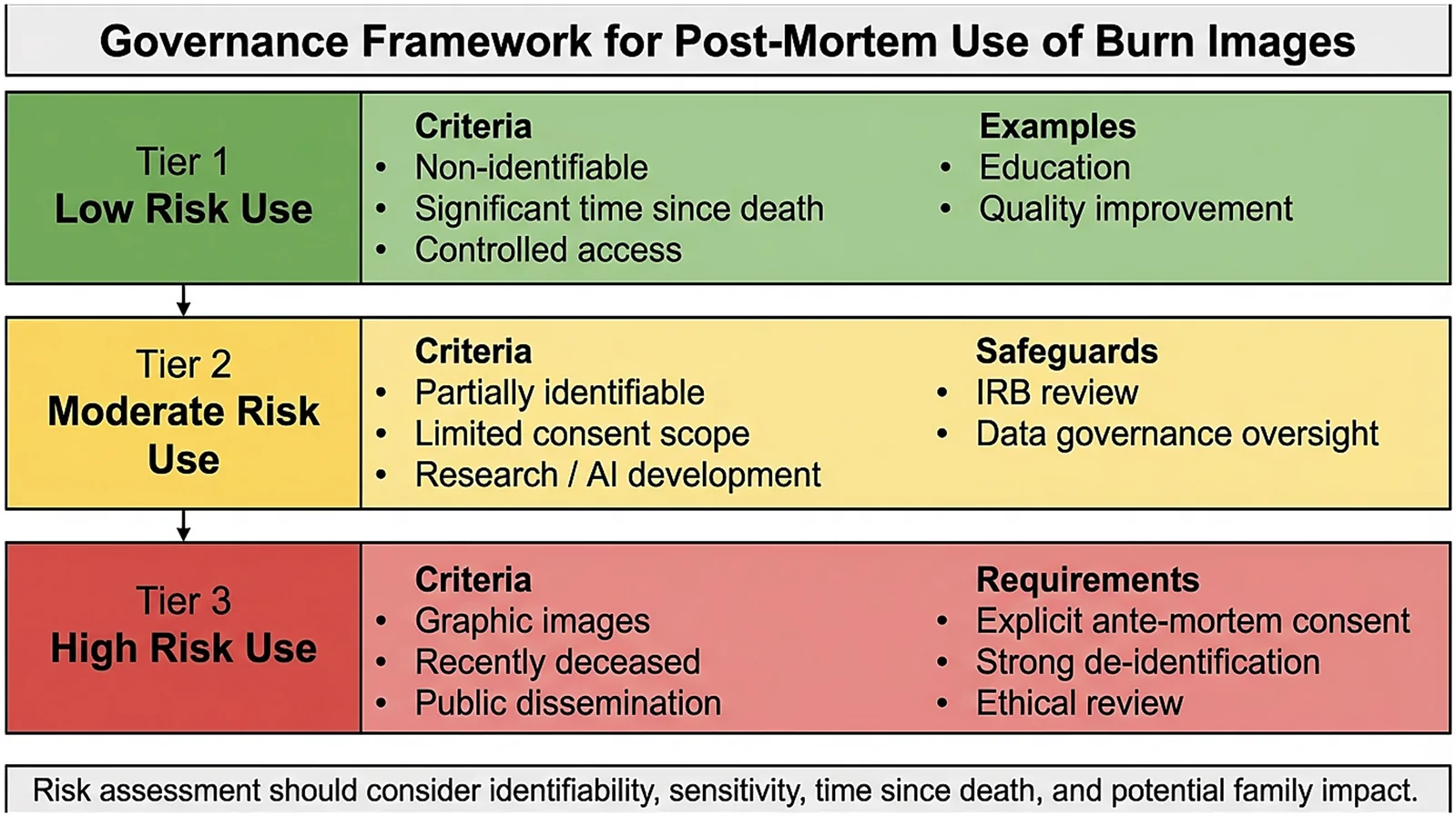
Governance Framework for Post-Mortem Use of Burn Images. Secondary uses of burn photographs are stratified by ethical risk, linking consent requirements and safeguards to image sensitivity, identifiability, and time since death. Image created by authors using FigureLabs.

The literature on PMDD and digital remains suggests several avenues for strengthening ethical governance. Rather than proposing a definitive governance model, Figure 2 organizes practical domains where the reviewed literature suggests current guidance is incomplete, including posthumous preferences, secondary use, identifiability, AI training, family interests, and public dissemination. These domains may serve as starting points for future empirical research, legal analysis, professional society guidance, and stakeholder consensus-building.

First, introducing structured opportunities for patients to express posthumous preferences about data and image use—analogous to organ donor registration or digital legacy planning—could help align practices with individual values.^47^ Simple additions to photography consent forms or advance care planning documents could invite patients to indicate whether they permit their burn images and records to be used after death for education, research, AI development, or public awareness campaigns, and whether they wish families to have a say in these decisions.^20^^,^^21^ While such directives would not resolve every conflict, they would provide ethically salient guidance and reduce the need for ad hoc judgments by clinicians or institutional review boards.

Second, contextual exceptionalism offers a framework for deciding when post-mortem use without explicit consent may nevertheless be ethically permissible. Bak and Willems argue that factors such as the sensitivity of the data, identifiability, cultural context, time since death, and the nature of the research question should shape decisions about when decedent privacy may be overridden in the public interest.^41^ Applied to burn images, this suggests that highly identifiable, graphic photographs of recently deceased patients should be used with extreme caution, perhaps only with explicit consent or under stringent de-identification and governance measures, whereas older, non-identifiable images may be ethically acceptable for inclusion in well-governed AI datasets with low re-identification risk.^18^^,^^41^ Such assessments require multidisciplinary deliberation, including input from burn clinicians, ethicists, legal counsel, data scientists, and, where possible, patient and family representatives. To clarify the practical relevance of these gaps, Table 2 summarizes common post-mortem uses of burn photographs and the unresolved ethical questions associated with each use, including consent, identifiability, family involvement, public dissemination, and AI-related secondary use.

**Table 2:** Three-Tier Governance Model for Post-Mortem Burn Photographs: Consent Threshold, Safeguards, and Dissemination Limits

**Use tier (typical use case)**	**Re-ID + dignity risk (low/mod/high)**	**Minimum consent + governance safeguards**	**References**
Tier 1: Internal education/QI (closed use)	Moderate (contextual identifiability; graphic content risk)	Prefer explicit image consent; restrict access to care team/learners; remove metadata; no onward sharing; secure storage and audit trail	^16^ ^,^ ^27^^,^ ^28^
Tier 2: Research + internal AI training (controlled access)	Moderate–High (linkage risk; secondary reuse; model persistence)	IRB review; purpose-specific consent when feasible; controlled-access repository; DUA; minimal metadata; periodic governance review; prohibit reuse outside approved scope	^33^ ^,^ ^34^^,^ ^38^^,^ ^42^
Tier 3: Public dissemination/open datasets/social media	High (uncontrolled spread; re-identification; family/community harm)	Avoid unless explicit, specific permission; strong necessity justification; no raw image release when distinctive features present; platform-aware consent; institutional oversight	^9^ ^,^ ^10^^,^ ^27^^,^ ^28^^,^ ^36^

Abbreviations: AI = artificial intelligence; DUA = data use agreement; IRB = institutional review board; QI = quality improvement; Re-ID = re-identification.

Third, the AI literature underscores the need to re-examine assumptions about de-identification. Evidence that facial structures and even non-facial features in imaging data can be re-identified when combined with public photographs or auxiliary information challenges the sufficiency of traditional anonymization methods.^13^ Burn photographs that reveal distinctive scarring patterns or tattoos may be similarly re-identifiable within specific communities, even if names and dates are removed.^48^ Ethical governance of post-mortem image use must therefore consider not only formal de-identification but also contextual identifiability and the potential impact of re-identification on families and communities.^41^ This may warrant limiting open release of raw burn image datasets and favoring controlled access repositories with clear data use agreements, audit trails, and oversight by ethics committees.

Finally, burn-specific ethics scholarship needs to catch up with these challenges. The 2018 review of ethics in burn care by Gerrek and colleagues identified a dearth of empirical and conceptual work, particularly from authors formally trained in clinical ethics, and called for closer collaboration between ethicists and burn clinicians.^22^ Subsequent work has deepened discussions of autonomy, treatment refusal, and end-of-life care, but has largely left data and image ethics unexplored.^23^^,^^45^ Given the centrality of photography, the rise of AI, and the growing attention to digital remains, burn societies and journals are well positioned to lead in articulating specialty-specific guidance. This could include consensus statements on clinical photography and AI training, model consent language that addresses post-mortem uses, and recommendations for respectful representation of deceased burn patients in educational and public materials.

## CONCLUSION

Burn care has become inseparable from extensive documentation and medical photography, yet the ethical status of these images and records after a patient’s death remains poorly defined. This narrative review shows that while general frameworks for post-mortem confidentiality and emerging scholarship on posthumous privacy and digital remains offer valuable conceptual tools, they have not been systematically applied to the distinctive context of burn care, where images are highly sensitive, technologically mobile, and increasingly used in AI development. The literature reveals consistent concerns about dignity, re-identification, and the lack of explicit patient directives, alongside recognition of the legitimate educational and research value of post-mortem data.

To align burn practice with evolving ethical concerns, future work by professional societies, institutions, journals, clinicians, ethicists, legal scholars, patients, and families is needed to determine whether additional norms are required and, if so, what form they should take. The findings of this review suggest that future guidance should consider posthumous privacy, patient preferences, image identifiability, AI-related secondary use, and respectful representation of deceased burn patients, while recognizing that this manuscript does not itself resolve those legal or ethical questions. By integrating concepts of posthumous privacy into everyday documentation, consent, and data governance, burn care can honor both the living and the dead while continuing to advance clinical knowledge and innovation.

## Notes

Author contributions: Joshua Khorsandi (Writing—original draft [lead]), Brian Mansoury (Writing—review & editing [equal]), Liahm Blank (Writing—review & editing [equal]), Abu-Bakr Ahmed (Conceptualization [lead]), Samir Alkhouri (Methodology [lead]), Kathryn Cataldo (Methodology [lead], Project administration [equal]), and Joshua MacDavid (Supervision [equal])

Funding: This research received no external funding.
